# Adapting mHealth Interventions to Improve Self-Management of HIV and Reduce Substance Use Among Emerging Adults in Zambia: Protocol for a Randomized Controlled Trial

**DOI:** 10.2196/99714

**Published:** 2026-07-17

**Authors:** Bo Wang, Karen MacDonell, Joseph Zulu, Ravi Paul, Ben S Gerber, Sylvie Naar, Jayasree Anitha Menon

**Affiliations:** 1 Department of Population and Quantitative Health Sciences University of Massachusetts Chan Medical School Worcestor, MA United States; 2 Center for Translational Behavioral Research Florida State University Tallahassee, FL United States; 3 School of Public Health University of Zambia Lusaka Zambia; 4 Department of Psychiatry University Teaching Hospital University of Zambia Lusaka, Lusaka Province Zambia; 5 School of Psychology Curtin University Dubai Dubai United Arab Emirates

**Keywords:** young people with HIV, HIV self-management, substance use, mobile health, mHealth, Healthy Choices, motivational text messaging, Zambia

## Abstract

**Background:**

Sub-Saharan Africa remains the region most affected by the HIV epidemic, with Zambia experiencing a substantial burden of disease. Young people with HIV are disproportionately impacted. Emerging adulthood (ages 18-24 years) is characterized by increasing independence, risk-taking behaviors, identity exploration, and changing social supports, all of which may impact HIV self-management, including medication adherence and retention in care. Substance use may further undermine HIV outcomes. Innovative interventions targeting HIV self-management and substance use reduction among Zambian young people are urgently needed.

**Objective:**

This study aims to develop and pilot a multicomponent mobile health (mHealth) intervention to improve HIV self-management and reduce substance use among young people in Zambia. We will adapt the 4-session in-person Healthy Choices intervention for mobile delivery (mobile Healthy Choices [mHC]) to increase access and delivery. We will also develop motivational text messaging (MTM) to enhance intervention impact. Both components will be delivered using the Computerized Intervention Authoring System. Using the multiphase optimization strategy (MOST) framework, we will assess preliminary efficacy and identify the most effective components.

**Methods:**

The study includes 3 phases. Phase 1 will involve conducting focus groups with Zambian young people with HIV to explore barriers and facilitators of HIV self-management and substance use to inform intervention adaptation. Phase 2 will involve adaptation and beta testing of mHC and MTM for functionality and feasibility using a community advisory board. Phase 3 will consist of a pilot trial using the MOST framework to evaluate feasibility, acceptability, and preliminary efficacy of intervention components. A total of 100 young people with HIV will be randomized to one of four experimental conditions: (1) standard antiretroviral therapy (ART) counseling (SAC), (2) mHC+SAC, (3) MTM+SAC, and (4) mHC+MTM+SAC. Primary outcomes will include feasibility, acceptability, ART adherence, viral suppression, and substance use. Feasibility and acceptability will be assessed through paradata of use patterns and the System Usability Scale. Participants will complete assessments at baseline and at 3- and 6-month follow-up visits. Biomarkers of ART adherence and/or sexually transmitted infections will also be collected.

**Results:**

Recruitment for this study began in May 2024 for phase 1. Focus group discussions were completed with 48 young people with HIV in July 2024. Phase 3, the pilot feasibility trial, began in June 2025. Upon project completion, we will have developed an innovative mHealth intervention to support HIV self-management and reduce substance use among Zambian young people with HIV.

**Conclusions:**

This study addresses a critical problem—suboptimal ART adherence, substance use, and challenges with HIV self-management—among young people with HIV in Zambia. We are developing 2 technology-based, theory-driven intervention components aimed at improving HIV self-management and substance use outcomes. If successful, this study will pave the way for scale-up of mHealth interventions to improve HIV care outcomes among young people with HIV in Zambia and other low-resource settings.

**Trial Registration:**

ClinicalTrials.gov NCT06415357; https://clinicaltrials.gov/study/NCT06415357

**International Registered Report Identifier (IRRID):**

DERR1-10.2196/99714

## Introduction

### Background

Despite significant advances in HIV prevention and treatment, HIV and AIDS remain a leading cause of morbidity and mortality in sub-Saharan Africa (SSA) [1]. In 2021, Zambia’s adult HIV prevalence (10.8%) was the seventh highest globally [1], with more than 15,000 new infections among youth aged 15 to 24 years [2]. Young people with HIV experience substantial disparities across the HIV care continuum, including lower antiretroviral therapy (ART) adherence, missed clinic visits, and lower viral suppression than older individuals [3,4]. Interventions designed for youth are crucial to improve adherence and viral suppression and to reduce mortality.

Young people with HIV are in emerging adulthood, a developmental stage marked by increasing independence, identity exploration, and risk-taking behaviors that may compromise HIV self-management [5]. Young people with HIV often exhibit poor HIV management, including suboptimal medication [6] and appointment adherence, limited engagement in health care [7], risky sexual behavior, alcohol use [8], and other substance use [9]. Zambian youth have the highest rate of regular alcohol use among 73 low- and middle-income countries [10] and the highest prevalence of cannabis use in Africa (37.2%) [11]. Alcohol use has synergistic and additive effects on HIV comorbidities [12] and accelerates HIV disease [13]. It is linked to increased HIV transmission [14], poor ART adherence [15], and worse outcomes across the treatment cascade [16]. In Zambia, commonly used substances include marijuana, cigarettes, opiates, cocaine, and inhalants [17], and 47% of youth have used a psychoactive substance [18].

Stigma is a well-documented barrier to health-seeking behaviors [19], engagement with the health care system [20], and adherence to treatment [21] across health conditions, including HIV [22]. For young people with HIV, stigma contributes to risky sexual behavior and substance use [23], reduces social support [24], and exacerbates mental health concerns such as depression [25]. Young people with HIV in Zambia widely experience HIV-related stigma across socioecological levels. Together, stigma and mental health are important targets for HIV interventions.

Strikingly, few interventions address self-management of both HIV and substance use in young people with HIV [26]. Healthy Choices (HC) is one of the few interventions that has demonstrated improvements in viral load and alcohol trajectories in young people with HIV [27,28]. HC also showed improvement in depression [29], marijuana use [28], and HIV-related stigma [30]. HC is a 4-session program that integrates motivational interviewing (MI) tailored for young people with HIV [27,31]. However, in-person HC can be limited by time, clinic engagement, and implementation barriers. Delivering HC via mobile health (mHealth) may increase access and engagement, reduce cost and time for youth and staff, and support implementation in clinics.

mHealth has been rapidly expanding across African countries, with widespread success in delivering health services [32]. Mobile connections in Zambia were equivalent to 91.4% of the total population in early 2022 [33]. SMS text messaging is inexpensive, nonintrusive, and widely integrated into the daily lives of youth in SSA [34]. Motivational text messaging (MTM) interventions have improved ART adherence and viral suppression among young people with HIV in SSA [35]. MTM offers a low-cost, sustainable, and youth-friendly way to extend the reach of HC sessions.

This study will adapt HC for mobile delivery and integrate MTM to improve HIV self-management and reduce substance use among young people with HIV in Zambia. It will develop and pilot a scalable, youth-friendly intervention to improve ART adherence, engagement in care, and health outcomes.

### Study Aims

We propose to develop and pilot a multicomponent mHealth intervention to improve HIV self-management and reduce substance use among young people with HIV in Zambia. First, we will adapt the 4-session HC intervention for mobile delivery and develop MTM to enhance the impact of mobile HC (mHC). Both components will be delivered using the Computerized Intervention Authoring System (CIAS). Second, we will conduct a pilot trial using the multiphase optimization strategy (MOST) framework to evaluate the feasibility, acceptability, and preliminary efficacy of intervention components and identify the most promising intervention component or combination of components. This study will lay the groundwork for a future fully powered efficacy trial and may provide a scalable model for improving outcomes among young people with HIV in Zambia.

### Theoretical Frameworks for Behavioral Change

**Figure 1:**
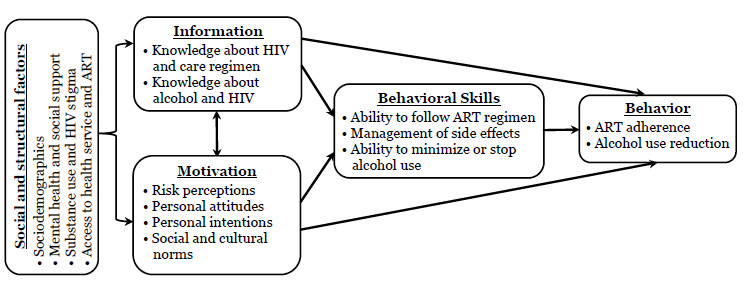
Integrated application of the information-motivation-behavioral skills model and the socioecological model for antiretroviral therapy (ART) adherence and alcohol use.

The information-motivation-behavioral skills (IMB) model and the socioecological model (SEM) will serve as the conceptual frameworks. Both theoretical models have been applied to HIV health behaviors [36] and ART adherence [37]. Although the IMB model focuses on individual-level determinants of behavior, the SEM captures the broader individual-, social-, and structural-level factors that influence HIV self-management and substance use. The proposed intervention focuses primarily on behavior change at the individual level while also addressing social- and structural-level influences, such as stigma, depression, and barriers to care engagement. According to the IMB model, behavior change results from the joint function of accurate information about risk behaviors (eg, risks of nonadherence to ART and substance use), motivation to change behavior, and behavioral skills to perform the behavior (Figure 1). According to the SEM, health behaviors are influenced by the complex interplay of multilevel factors related to these elements. We will develop intervention content to address both individual and multilevel influences to facilitate HIV self-management and reduction of substance use among young people with HIV in Zambia.

### Intervention Components

#### Mobile Healthy Choices

mHC is a 4-session computer-delivered intervention. CIAS supports MI-based intervention delivery by allowing participant choices and branching to tailored content throughout the session. For instance, a participant indicating high levels of motivation for improving HIV management will be routed to different content than a participant with lower motivation. During sessions, specific strategies to overtly communicate acceptance and support autonomy are included to address stigma and strengthen motivation for change. In session 1, young people with HIV choose 1 of 2 primary target behaviors (HIV self-management or alcohol use). Young people with HIV will focus on the selected behavior, though other behaviors such as sexual risk, stigma, depression, and other substance use will be addressed during sessions. The virtual counselor (avatar) will use standard MI techniques to elicit the participant’s perspective on the selected problem and build motivation for change by eliciting and reinforcing change talk. The avatar will deliver feedback based on responses to prompts within the session and discuss a behavior change plan, and the youth will set the change plan goal. The second session follows the same format for the second behavior. In sessions 3 and 4, the avatar will review the change plan, reinforce motivation and confidence, address barriers through problem-solving, consolidate commitment, and discuss strategies to maintain behavior change [38]. Sessions will also address contextual issues, including stigma.

#### MTM

SMS text messages will consist of motivational statements promoting self-management of HIV and reductions in alcohol use and will be developed based on findings from phases 1 and 2. CIAS will be used to automate and tailor MTM delivery. The system will allow flexibility in message timing and frequency and will automatically record message delivery status. MTM will be sent daily for 2 months, and weekly for an additional 4 months (months 3-6) at times chosen by the participants. MTM content will be tailored on readiness to change, as assessed during mHC session 1. Participants will be informed that mobile phones may be shared or accessed by others and will be advised to review study-related messages only in private settings. All SMS text messages will use neutral, nonidentifying language and will avoid explicit references to HIV, substance use, or mental health concerns. Participants may discontinue MTM at any time. Research staff will be trained to recognize participant distress, substance use concerns, and potential safety risks; participants requiring additional support will be referred to appropriate HIV, mental health, or substance use treatment services available through collaborating clinical sites. All MTM content will be reviewed by the community advisory board (CAB) before trial launch to ensure cultural appropriateness, clarity, and privacy protection.

#### Standard ART Counseling

All participants will receive one-on-one, in-person counseling at a clinic concerning the behavioral, psychosocial, and medical implications of HIV. This standard counseling session will last approximately 30 minutes. Health care providers connect newly diagnosed individuals to a range of services, including substance use treatment, mental health care, reproductive counseling, risk-reduction interventions, case management, and partner services. Clients also receive counseling on ART adherence and the management of treatment-related side effects.

## Methods

### Study Setting

Young people with HIV will be recruited from 2 large HIV clinics in Lusaka Province to ensure sample diversity. Lusaka is home to 3.4 million people (18.8% of Zambia’s population) [39] and has high HIV rates and high phone ownership among youth [40]. The Kanyama First Level Hospital (KFLH) serves a population of approximately 120,000 individuals aged 10 to 29 years in a high-density, low-income township near Lusaka [41]. The Matero Urban Health Centre (MUHC) is located in urban Lusaka and serves a population of 247,451 [41]. We will recruit young people with HIV from a broad range of socioeconomic backgrounds. MUHC typically serves middle- to higher-income families, whereas KFLH primarily serves lower-income communities. Together, these clinics provide access to diverse youth populations with substantial HIV burden and co-occurring substance use.

### Study Design

There are 3 phases in the study: phase 1 (formative research), phase 2 (intervention adaptation), and phase 3 (pilot randomized controlled trial [RCT]).

#### Phase 1: Formative Research

##### Overview

The first phase of the assessment, decision, adaptation, production, topical experts-integration, training, testing framework [42] is a needs assessment of the target population to guide cultural and developmental tailoring through 6 focus groups with young people with HIV in Lusaka. Focus groups are commonly used to inform intervention adaptation for young people with HIV in Africa, including Lusaka [43]. Cultural adaptation will be led by the Zambian and US investigators, who have expertise in working with young people with HIV, substance use, and mHealth intervention adaptation. We will conduct separate focus groups for male- and female-identifying young people with HIV (3 each), with 6 to 8 participants per group, using best practices for youth [44]. Focus group guides will be developed by the study team and structured around the IMB model. Topics will include knowledge about HIV self-management and substance use (information); attitudes toward ART, risk perceptions, and reasons for taking ART and attending appointments (motivation); and facilitators or barriers to ART adherence, appointment attendance, , and substance use (behavioral skills). We will also explore social supports, past successes, and strategies for overcoming barriers. Responses will be used to inform intervention content and delivery, including privacy concerns, disclosure, and acceptable session length. Participants will be recruited from MUHC (N=20) and KFLH (N=20) using purposive sampling to ensure representation. Focus groups will be audiotaped, transcribed, and will last approximately 1 to 1.5 hours.

##### CAB

A CAB of key stakeholders will be identified and assembled by the Zambia investigators, who have developed strong relationships with health care providers serving young people with HIV in Lusaka through years of HIV research. The CAB will include 4 young people with HIV, 4 Zambian health personnel (physicians, nurses, or other personnel engaged in direct care of young people with HIV), and 2 community leaders (eg, people living with HIV, advocates, nongovernmental organization representatives, or HIV researchers). Members will meet quarterly, with additional meetings as needed. The CAB will provide iterative feedback on HC and MTM intervention content, study materials, recruitment and retention strategies, privacy, stigma, cultural relevance, and implementation procedures for young people with HIV in Zambia. The first meeting will establish CAB functions and procedures consistent with community-based participatory research principles. Any inconsistencies in feedback will be resolved during meetings or, if needed, through discussion with the Zambia-US study team. Detailed notes will be taken during meetings. Study materials and intervention approaches will be revised based on the CAB’s feedback.

#### Phase 2: Adapting and Refining Motivational Enhancement System for Preexposure Prophylaxis Uptake and Adherence and MTM

##### Adapting HC for mHealth to Target Self-Management of HIV and Substance Use

Formative research will provide rich data on substance use and HIV self-management among young people with HIV within the Zambian cultural context and inform intervention development. We will use an established approach that progresses from formative research to pilot adaptation and RCT testing, guided by a framework for HIV interventions. The intervention flow is based on MI and progresses from exploration of the importance of target behaviors and confidence in these behaviors to goal setting. Participants will explore ambivalence around HIV self-management and reducing substance use, as well as barriers they may encounter, strategies they may use, and available social supports. Components will be adapted based on findings from phase 1. Zambian investigators will translate materials into Bemba and Nyanja. The HC adaptation process will involve initial programming in collaboration with the CAB and evaluation of the intervention content and its cultural acceptability through a youth advisory board and beta testing.

##### Developing and Programming MTM

MTM will be developed using phase 1 focus groups and delivered by CIAS to support HIV self-management and substance use reduction. Peer messages will be developed using a cowriting strategy [45]. We will use this collaborative, community-based approach to build the database for this study by including peer messages written by Zambian young people with HIV selected from the focus groups. First, young people with HIV will be asked to describe 3 to 5 factors or situations that have facilitated or challenged their efforts to improve HIV self-management and reduce substance use, as well as strategies used to overcome barriers. Next, for each scenario described, they will be asked to write messages to help other young people with HIV in a similar situation. To ensure coverage of key topics, the co-writing strategy [45] will focus on developing messages that educate and motivate participants regarding HIV self-management, substance use reduction, HIV-related stigma, and other themes identified during the group discussions. Our team will write messages for these topics and ask young people with HIV to rewrite them in their own words, which will then be reviewed by the CAB. MTM will be sent daily for 2 months, followed by weekly for 4 months at a time of the participant’s choosing to promote autonomy and engagement.

##### Prepilot Testing of mHC and MTM

The final step of assessment, decision, adaptation, production, topical experts-integration, training, testing is testing, conducted in 2 steps: theater testing and one-on-one beta testing. In step 1, theater testing pretest methodology172 will be used with the CAB to gauge participants’ reactions to the mHC and MTM components. During theater testing, facilitators will demonstrate excerpts of the mHC sessions and MTM that capture core elements of the intervention. At the end of each excerpt, CAB members will complete brief surveys that contain closed-ended and open-ended questions to elicit their reactions regarding the appropriateness of the elements for the new population. The goal is to collect critiques of the material, content, and delivery and to identify changes that should be included to enhance relevance and efficacy for the target population.

Following modification based on step 1, step 2 will involve systematic one-on-one beta testing over 2 rounds of 5 participants from the target population (10 in total), staggered to allow iterative feedback on technological components and intervention content, followed by refinement. During each round, young people with HIV will complete 4 HC sessions within 2 months, as well as daily MTM between HC sessions. We will also administer baseline and postintervention measures in the beta study to obtain feedback on readability and participant burden. Moreover, 24 hours after the first MTM, staff will call the participant to check for any issues. After completing all mHC sessions, participants will receive an SMS text message prompting them to complete a brief online survey. Participants who do not respond will be contacted by telephone. The survey will collect feedback on technical issues and participants’ experiences with session length, delivery, content, and the receipt and comprehension of MTM. An interview will be conducted after all the sessions are completed to further assess feasibility and acceptability: (1) review technical or other issues in delivering components and (2) solicit input on how to refine delivery and content. mHC and MTM will be modified before round 2, which will follow the same format as round 1. Following theater testing and 2 rounds of beta testing, we expect the refined intervention components to be ready for the pilot trial.

#### Phase 3: Pilot MOST Design–Based Trial

##### Overview

We will evaluate the feasibility, acceptability, and preliminary efficacy of adapted intervention components to improve HIV self-management and substance use in a MOST design–based trial (ClinicalTrials.gov NCT06415357). The design is shown in Table 1. The trial will examine the effect of each component and whether the presence or absence of a component has an impact on the performance of other components. An advantage of the MOST framework is its ability to identify intervention components that make significant contributions to overall effectiveness and should therefore be retained in an optimized intervention package. We will use a full factorial design because of its relative simplicity and cost-efficiency [46]. For our trial, there are 2 components, each with 2 levels, resulting in a 2×2 full factorial design with 4 experimental conditions (Table 1). We will use viral suppression as a measure of success (viral load <200 copies/mL) [47] and optimization criterion. We will enroll 100 young people with HIV who are not adherent to ART and report unhealthy alcohol use [48] or any drug use. “Standard ART counseling” will be provided to all participants as a constant component. Young people with HIV will be randomly assigned to 1 of 4 conditions (Table 1). The primary outcome will be ART adherence and HIV viral load. The main effects of the components and interactions between them will be estimated using ANOVA. The goal of this pilot trial is to estimate preliminary effect sizes, evaluate feasibility and acceptability, and identify promising intervention components for inclusion in a future fully powered RCT. This protocol is developed in accordance with the SPIRIT (Standard Protocol Items: Recommendations for Interventional Trials) guidelines (Multimedia Appendix 1).

**Table 1:** Full factorial design with 4 experimental conditions.

Experiment conditions	Standard antiretroviral therapy counseling	Mobile Healthy Choices	Motivational text messaging
1 (n=25)	Yes	Yes	Yes
2 (n=25)	Yes	Yes	No
3 (n=25)	Yes	No	Yes
4 (n=25)	Yes	No	No

##### Randomization to Conditions

We will use covariate-adaptive randomization [49] to achieve balance on key baseline confounding variables across 4 experimental conditions. Specifically, the project manager will conduct covariate-adaptive randomization after the baseline interview. Key information on participants (eg, gender, substance use behavior, and level of ART adherence) will be extracted at recruitment and will be used as input data for a SAS randomization program created by the UMass Quantitative Methods Core.

### Study Sample

Young people with HIV will be included if they provide informed consent. Inclusion criteria will be as follows: (1) living with HIV and aged between 18 years 0 months and 24 years 11 months; (2) visual analog scale showing <80% medication adherence in the last month and unhealthy alcohol use or any drug use in the last month [50]; and (3) able to understand, read, and speak English, Nyanja, or Bemba. Exclusion criteria are as follows: (1) a serious cognitive or psychiatric problem that would compromise ability to provide informed consent and (2) current enrollment in another HIV intervention study. Smartphone ownership is not required; a smartphone with internet access will be provided at no cost to participants who do not have one.

### Sample Size of the Trial

This pilot MOST design–based trial is designed to estimate main effects of mHC and MTM and their interaction using a 2×2 factorial study design. Using G*Power 3.1.9 (ANOVA procedure), with effect size f=0.35, α=.05, and sample size=100 (25 per condition), statistical power is 80% for detecting main effects of mHC and MTM and 73% for detecting their interaction. Our proposed pilot trial is not an efficacy study, but our sample is large enough to detect a medium to large effect size of mHC and MTM.

### Recruitment and Enrollment Procedures

Youth presenting at either clinic will be screened by the clinic staff for eligibility and, if applicable, referred to research staff for informed consent. After providing verbal consent, the research staff will determine eligibility. Screening, enrollment, and baseline assessment will occur on the same day whenever possible; otherwise, they will be completed within 1 week. After written consent, participants will be asked to provide a phone number or email and contact information for a family member and/or friend in case they cannot be reached.

### Data Collection Procedures

Table 2 summarizes the study measures, including primary and secondary or exploratory outcomes. Self-report measures will be administered using cloud-based computer-assisted self-interviewing at baseline and 3- and 6-month follow-up. Blood samples for dried blood spot (DBS) testing for HIV viral load will be collected at baseline and 3 and 6 months. Sexually transmitted infection outcomes, including syphilis, gonorrhea, and chlamydia, will be assessed at baseline and 6 months. In computer-assisted self-interviewing, participants view each question and response options on a private iPad (Apple Inc) screen and enter responses directly into password-protected Qualtrics (Qualtrics).

**Table 2:** Study measures.

Measures		Scale or description
**Primary outcomes**		
	Acceptability	System Usability Scale, Client Satisfaction Questionnaire, and exit interviews
	Feasibility	Retention rate ≥85% at 6-month follow-up, number of responses to SMS text messages, and number of HC^a^ sessions completed
	ART^b^ adherence	Visual analog scale, 4-week percent of doses taken, and DBS^c^ for HIV viral load (viral suppression defined as <200 copies/mL)
	Substance use	ASSIST^d^ and AUDIT-C^e^
**Secondary outcomes**		
	IMB^f^ constructs-information	Knowledge of HIV transmission risk and ART knowledge
	IMB constructs-motivation	Behavioral intentions and attitudes toward risk behaviors
	IMB constructs-behavioral skills	Perceived efficacy as a proxy measure of behavior skills
	STI^g^	Syphilis, gonorrhea, and chlamydia
	Sexual risk	Timeline followback: sexual behaviors in the past 30 days
**Secondary and exploratory—contextual factors**		
	Mental health	Brief Symptom Inventory
	Social support	Social Provision Scale
	HIV stigma	Shortened version of the Berger Stigma Scale

^a^HC: Healthy Choices.

^b^ART: antiretroviral therapy.

^c^DBS: dried blood spot.

^d^ASSIST: Alcohol, Smoking, and Substance Involvement Screening Test.

^e^AUDIT-C: Alcohol Use Disorders Identification Test–Consumption.

^f^IMB: information-motivation-behavioral skills.

^g^STI: sexually transmitted infection.

The primary acceptability outcome will be measured by the System Usability Scale, a 10-item Likert scale assessing usability. A score of >70 indicates that technology-based interventions are acceptable [51]. We will also assess interest in future use of the intervention at study completion. For feasibility, we will assess responses to MTM, number of mHC sessions completed, cumulative time spent in mHC, and recruitment rates. Preliminary efficacy outcomes include ART adherence and alcohol use. ART adherence will be measured using (1) the Young Adult Adherence Interview with a visual analog scale [52], (2) 4-week percent of doses taken, and (3) DBS biological testing. Results of DBS testing will be triangulated with self-reports. We will assess integrated behavior model constructs, including knowledge, attitudes, motivations, and behavioral skills related to taking ART, and important contextual factors (depression and HIV stigma).

### Data Analysis

We will estimate retention rates overall and per condition and examine differences across conditions using chi-square tests. We will assess differences in acceptability using 2-tailed *t* tests or Mann-Whitney *U* tests. Minimum criteria for acceptability and feasibility will include a mean System Usability Scale score ≥70 and at least 50% of participants responding to MTM at least once and completing at least 2 mHC sessions [51].

To test the main effects and interactions between components using data from the 2×2 factorial study design, we will use ANOVA with effect coding (−1=“no” condition; +1=“yes” condition) rather than dummy coding. The preliminary intervention effects on ART adherence and substance use outcomes will be assessed using linear mixed-effects models, while viral suppression will be analyzed using mixed-effects logistic regression models. Models will adjust for baseline values of the outcome and relevant covariates and will account for repeated assessments over time. Missing data will be handled using full information maximum likelihood estimation. Effect sizes will be estimated for the primary outcomes.

We will make a preliminary selection of components that have achieved main effects. This selection will then be re-evaluated considering any substantial interaction effects to understand how the components work in combination. Depending on the optimization criterion (viral suppression), this will be combined with other information (eg, cost, feasibility, and scalability) to make a final selection of components. This information will inform the development of an optimized intervention package.

### Ethical Considerations

This protocol was reviewed and approved by the institutional review boards of the University of Massachusetts Chan Medical School in the United States (STUDY00001496) and the University of Zambia (4548-2023).

## Results

Recruitment for this study began in May 2024 for phase 1. Qualitative interviews were completed with 48 young people with HIV in July 2024. Qualitative data analysis has been completed, and the results were used to inform intervention adaptation and refinement. In phase 2, our team developed 4 MTM libraries focused on (1) ART adherence, (2) substance use, (3) self-management, and (4) empowerment, containing 76 messages; these messages were reviewed by the youth advisory board and investigator team for refinement. Refined MTM will be delivered to participants through SMS 2.0, an SMS text messaging platform. Our team also developed a 4-session intervention (mHC) to promote ART adherence and substance use reduction using the CIAS (version 3.0). Ten Zambian young people with HIV completed one-on-one beta testing of the intervention components and provided additional feedback to guide refinement. Phase 3, the pilot feasibility trial, began in June 2025. The findings will be disseminated to stakeholders and communities of interest through peer-reviewed journals, academic conferences, and other communication channels.

## Discussion

### Expected Outcomes

We anticipate developing a multicomponent mHealth intervention to improve ART adherence and HIV self-management and to reduce substance use among young people with HIV in Zambia. young people with HIV in Zambia often have difficulty taking medication as prescribed, miss medical appointments, and report unhealthy alcohol and other drug use, which may compromise viral suppression and long-term health outcomes. Combining mHC and MTM may help improve adherence, strengthen self-management skills, and reduce substance use among Zambian young people with HIV.

We will evaluate the feasibility, acceptability, and preliminary efficacy of an mHealth intervention combining mHC and MTM. On the basis of our theoretical models of behavior change, we anticipate that the intervention will be beneficial, although this remains to be determined. If successful, this intervention can improve the health of those affected by HIV or AIDS and reduce future rates of HIV infection in Zambia. Our research is integrated within Zambia’s national HIV prevention effort. The commitment and ongoing involvement of the University of Zambia will allow this research to serve as a model for HIV prevention among high-risk groups, especially in SSA and other low-resource settings.

### Planned Next Steps

The project will be conducted to develop an optimized technology-based intervention for young people with HIV in Zambia and prepare for a future large-scale RCT. For a fully powered R01 project, we propose a multisite study testing the efficacy of mHC and MTM using a type 2 effectiveness-implementation design, with testing of intervention effectiveness in improving ART adherence, viral suppression, and substance use outcomes, while gathering information on implementation, including cost analysis to assess the relative cost of implementing the intervention.

### Conclusions

This study addresses a critical problem—suboptimal ART adherence, substance use, and challenges with HIV self-management—among young people with HIV in Zambia. We are developing 2 potentially synergistic, technology-based, theory-driven intervention components aimed at improving HIV self-management and substance use outcomes. Despite major advances in HIV treatment, many young people with HIV continue to face barriers to sustained adherence and viral suppression. Our study addresses these barriers through a youth-centered mHealth approach. It is responsive to the need for scalable interventions to improve HIV outcomes. If successful, this study will pave the way for the scale-up of mHealth interventions to improve HIV care outcomes among young people with HIV in Zambia and other low-resource settings. The NIH peer review summary statement is provided in Multimedia Appendix 2.

## Appendix

Multimedia Appendix 1 SPIRIT checklist.

Multimedia Appendix 2 Peer-reviewer report from IPTA -Interventions to Prevent and Treat Addictions Study Section, Risk, Prevention and Health Behavior Integrated Review Group (National Institutes of Health, USA).

## Abbreviations

ART: antiretroviral therapy
CAB: community advisory board
CIAS: Computerized Intervention Authoring System
DBS: dried blood spot
HC: Healthy Choices
IMB: information-motivation-behavioral skills
KFLH: Kanyama First Level Hospital
mHC: mobile Healthy Choices
mHealth: mobile health
MI: motivational interviewing
MOST: multiphase optimization strategy
MTM: motivational text messaging
MUHC: Matero Urban Health Centre
RCT: randomized controlled trial
SEM: socioecological model
SSA: sub-Saharan Africa

## Data Availability

The data generated during this study are available from the corresponding author upon reasonable request.
