# Supplementary material for: Adapting mHealth Interventions to Improve Self-Management of HIV and Reduce Substance Use Among Emerging Adults in Zambia: Protocol for a Randomized Controlled Trial
Source: JMIR Res Protoc. 2026 Jul 17;15:e99714. doi: 10.2196/99714 (PMC13428203; doi:10.2196/99714)
Supplement: Multimedia Appendix 2 [file resprot_v15i1e99714_app2.pdf]

SUMMARY STATEMENT

(Privileged Communication)

PROGRAM CONTACT:
Guifang Lao
301-443-1061
laog@mail.nih.gov

Release Date: 07/13/2023
Revised Date: 07/13/2023

Application Number: 1R34DA059935-01

Principal Investigators (Listed Alphabetically):

MACDONELL, KAREN
WANG, BO (Contact)

Applicant Organization: UNIV OF MASSACHUSETTS MED SCH WORCESTER

Review Group: IPTA
Interventions to Prevent and Treat Addictions Study Section
AIDS - EXP. REV.

Meeting Date: 06/22/2023
Council: OCT 2023
Requested Start: 12/01/2023
Opportunity Number: PAR-22-183
PCC: MM/GLA

Project Title: Adapting mHealth interventions to improve self-management of HIV and substance use among emerging adults in Zambia
SRG Action: Impact Score:16
Next Steps: Visit https://grants.nih.gov/grants/next_steps.htm
Human Subjects: 30-Human subjects involved - Certified, no SRG concerns
Animal Subjects: 10-No live vertebrate animals involved for competing appl.
Gender: 1A-Both genders, scientifically acceptable
Minority: 5A-Only foreign subjects, scientifically acceptable
Age: 7A-Only Adults, scientifically acceptable

Project Year	Direct Costs Requested	Estimated Total Cost
1	150,000	224,659
2	150,000	224,659
3	150,000	224,659
TOTAL	450,000	673,978

ADMINISTRATIVE BUDGET NOTE: The budget shown is the requested budget and has not been adjusted to reflect any recommendations made by reviewers. If an award is planned, the costs will be calculated by Institute grants management staff based on the recommendations outlined below in the COMMITTEE BUDGET RECOMMENDATIONS section.

1R34DA059935-01 Wang, Bo

RESUME AND SUMMARY OF DISCUSSION: This application proposes to use the Multiphase Optimization Strategy (MOST) framework to assess the feasibility, acceptability, and preliminary efficacy of Mobile Healthy Choices (mHC) and Motivational Text Messaging (MTM) on antiretroviral therapy (ART) and substance use among young people with HIV. During discussion, the panel noted the study's high significance in addressing a critical gap in research and clinical practice on HIV and substance use treatment in a high-risk and high-need group of young people through a culturally tailored intervention that simultaneously addresses both HIV medication adherence and substance use, with the potential for a large scale dissemination. The use of artificial intelligence software and technology to develop the platform for the intervention was considered innovative. The investigative team is excellent with requisite expertise, experience, and a demonstrated history of collaboration. The environment is outstanding with adequate supports and resources, including strong partnerships with local communities in Zambia. Additional strengths noted underscore the rigor of the approach, including thoughtful adaptation of the intervention, data collection methods, and analytic plan. Very few, minor, and addressable weaknesses were identified, including different treatment delivery for the intervention (via mobile phone) and control (in-person) conditions; insufficient description of the control group; and insufficient rationale for asking intervention participants to choose from either HIV self-management or substance use reduction versus both. Following the discussion, the panel agreed that the study is expected to have a high impact in advancing and guiding research and clinical practice for improving HIV self-management and reducing substance use among young people with HIV in Zambia.

DESCRIPTION (provided by applicant): Sub-Saharan Africa is most severely affected by the HIV epidemic, particularly Zambia. Young people with HIV (YPWH) are disproportionately impacted by HIV/AIDS. Emerging adulthood (ages 18-24) is characterized by increasing independence, risk-taking behaviors, identity exploration, and changing social supports, which may impact HIV management behaviors, including medication adherence, retention in care, sexual risk behavior, and substance abuse. Substance use has synergistic and additive effects on health and HIV-related comorbidities, and accelerated HIV disease. Self-management interventions targeting HIV and substance use reduction in Zambian YPWH are urgently needed. We propose to develop and pilot a multi-component, mHealth intervention to promote HIV self-management and reduce substance use. We will adapt the 4- session, in-person Healthy Choices (HC) intervention for mHealth (mHC) to increase access and delivery of HC. In addition, we will develop and pilot test Motivational Text Messaging (MTM) to enhance the impact of mHC. mHC and MTM will be delivered using Computerized Intervention Authoring System (CIAS). Our primary aim is to develop and assess the preliminary efficacy of the resulting mHealth intervention. We will utilize a Multiphase Optimization Strategy (MOST) framework to identify the most effective intervention component or combination of components that addresses self-management and substance use among YPWH in Zambia. The proposed study includes 3 phases. Phase I includes focus groups with Zambian YPWH to explore barriers and facilitators of HIV self-management and substance use to inform the intervention. Phase II consists of adapting and beta testing of mHC and MTM for functionality and feasibility using a community advisory board of Zambian YPWH, local healthcare personnel, and community leaders. In Phase III, we will conduct a pilot using the MOST framework to assess feasibility, acceptability, and preliminary efficacy of intervention components (mHC and MTM) to improve HIV self-management and reduce alcohol and other drug use. One hundred YPWH be randomized to four experimental conditions: 1) Standard ART Counseling (SAC), 2) mHC + SAC, 3) MTM + SAC, and 4) mHC + MTM + SAC. Feasibility and acceptability of the intervention will be assessed through paradata of usage patterns and the System Usability Scale. Preliminary impact will be assessed by evaluating ART Adherence and substance use. YPWH will complete assessments at baseline and 3-, and 6-months post-intervention. Biomarkers of adherence to ART and HIV/STI will be

collected. Upon project completion, we will have an optimized mHealth intervention to support YPWH's self- management of HIV which will be ready for testing in a larger efficacy trial.

PUBLIC HEALTH RELEVANCE: Young people with HIV in Zambia often have difficulty taking medication as prescribed, miss medical appointments, and drink excessive alcohol and use other drugs. This project will develop and implement a multi-component, mHealth intervention designed to improve HIV self-management and reduce substance use. If successful, this intervention can improve the health of those affected by HIV/AIDS and reduce future rates of HIV infection in Zambia.

CRITIQUE 1

Significance: 1
Investigator(s): 1
Innovation: 2
Approach: 2
Environment: 1

Overall Impact: This is a highly significant application addressing critical, intractable and interconnected problems - alcohol use and HIV self-management behaviors - among emerging adults (ages 18-24) in a Sub Saharan African (SSA) country, Zambia. The project's significance is underscored by Zambia's exceptionally high HIV prevalence rate and by the population targeted for the intervention: Zambian emerging adults have a high risk of HIV exposure and (like emerging adults worldwide) are resistant to engaging in and adhering to available treatments. Additionally, there are lack of appropriately tailored interventions targeting both of these issues simultaneously (HIV and alcohol/drug use). The feasibility of the ambitious plan to culturally adapt and ultimately launch a pilot RCT of electronically delivered, theoretically motivated interventions (Healthy Choices and Motivational Text Messaging) in a factorial design is supported by the highly experienced and accomplished investigative team that includes a group of key collaborators on the ground at the University of Zambia, experts in technologically adapted motivational interventions for youth, and experienced investigators who have implemented complex interventions with high risk youth in other countries. The investigators have structured the application to ensure that contextual factors will be accounted for in the intervention (text messaging) with the initial focus group activities. They have also provided clear and feasible criteria for determining participant eligibility. Minor concerns is that the design relies completely on technology and on the cooperation of participants (emerging adults) who may be difficult to recruit and retain; additionally there are no objective indicators of substance use. Given the careful and thorough structure of the application which includes solid plans for feasibility assessment and for understanding barriers to implementation, overall, this application has a very high potential for informing impactful interventions with high-risk emerging adults.

1. Significance:

Strengths

- Sub-Saharan Countries in general, and particularly Zambia have been hit hard by the HIV epidemic as indicated by their global prevalence ranking.
- The spread of the virus is particularly problematic among adolescents and emerging adults due to low medication adherence and lack of retention in HIV care.
- High risk emerging adults in SSA have low viral load suppression.

- Exceptionally high rates of alcohol and marijuana use among Zambian youth potentially further fuels their risk of spreading the virus.
- Partially due to stigma related to socio-cultural norms, Zambian HIV positive youth face a lack of treatment options.
- Again, partially due to socio-cultural norms Zambian youth are resistant to widely available treatment, face challenges to screening for HIV, engaging in HIV treatment, and adhering to HIV medications.

Weaknesses

- None.

2. Investigator(s):

Strengths

- An exceptionally strong MPI team with a history of collaboration and complementary expertise.
- The corresponding PI, Dr. Wang, who is a behavioral epidemiologist and biostatistician based at the Mass. Medical Center, has expertise in HIV risk prevention, adolescent health risk behavior, and implementation of mhealth interventions for youth; Dr. Wang has experience using the MOST framework proposed here; he has considerable experience developing international collaborative interventions.
- MPI MacDonnell has expertise in mhealth interventions, interventions specifically targeted toward adolescents at risk, and interventions addressing HIV and alcohol use. She brings strength to the team as a developmental psychologist who is familiar with studying emerging adults. Further, her successful implementation of mobile phone-based text messaging and computer delivered motivational interview – based interventions enhances the feasibility of this application.
- Co-I Gerber adds expertise in health services research with mHealth interventions (especially in the areas of motivational text messaging).
- The team includes three scientists as co-investigators who are based at the University of Zambia: Drs. Paul , Menon, & Zulu. This team of SSA-based collaborators bring unique scientific and clinical strengths that should support successful adaptation and implementation of the intervention.
- The developer of the Healthy Choices intervention, Dr. Naar, is a consultant on the study and adds further expertise in the application of MI to changing health behavior in youth.

Weaknesses

- None.

3. Innovation:

Strengths

- Use of the NIH-developed Computerized Intervention Software (CIAS) to tailor individual motivational text messages is innovative.
- The simultaneous focus on HIV self-management and substance use using a culturally and developmentally tailored mhealth approach is also innovative.
- Use of the MOST framework to guide intervention development is also somewhat innovative.

- The decision to focus HIV prevention/intervention efforts on high-risk substance involved emerging adults in SSA is in and of itself highly innovative and unique.

Weaknesses

- None.

4. Approach:

Strengths

- The intervention development proceeds in a logical manner.
- The beta testing and feedback phases (phases one and two) facilitate critical adjustments/changes/adaptations to intervention content.
- The incorporation of a CAB with diverse stakeholders – including youth and community members - to advise project development is a strength.
- The intervention is guided by appropriate theory (Information/Motivation/Behavior).
- The plan to use Healthy Choices and Motivational text messaging as key intervention components in a trial is informed by preliminary research on efficacy among US youth.
- The use of CIAS to deliver the mHealth messaging (MTMs) and adapt software content is a strength.
- Use of blood spots for assessing viral load is a strength.
- Screening criteria for selection into the study (i.e., lack of adherence, unhealthy alcohol use and any drug use) is clearly operationalized and feasible.
- Contextual variables that may be impacting decision making and adherence are assessed and accounted for in the formative phase of the research (focus groups).

Weaknesses

- There are no objective indicators (biomarkers) of substance use incorporated into the study.
- In the RCT, the intervention tested is completely online via mobile phone; yet the control condition (SAC) is completely in person; this is a minor weakness as alternative strategies may be less feasible/practical.
- Drop out/retention is a critical issue in studies employing emerging adults; the plan to address this is weak (although it is noted by the applicant as a potential problem).

5. Environment:

Strengths

- University of Massachusetts Medical School, where the contact PI is based and Florida State University, where the other PI is based, have excellent resources to support the completion of the study.
- The University of Zambia, where the SSA collaborators are located, has excellent resources for on-site study development and completion.
- The team has access to two key clinics that will serve as the basis for participant recruitment, further underscoring the strength of the environment.

Weaknesses

- None.

Study Timeline:

Strengths

- Project divided into 3 phases over 36 months with a logical plan for activities moving towards the pilot RCT in the final phase.
- The team has experience implementing studies targeting high risk populations in other countries supporting the feasibility of this ambitious timeline.

Weaknesses

- May be somewhat ambitious.

Protections for Human Subjects:

Acceptable Risks and/or Adequate Protections

- Human subject projections are adequate.

Data and Safety Monitoring Plan (Applicable for Clinical Trials Only):

Acceptable

- o They will form an appropriately constructed external DSMB.

Inclusion Plans:

- Sex/Gender: Distribution justified scientifically
- Race/Ethnicity: Distribution justified scientifically
- For NIH-Defined Phase III trials, Plans for valid design and analysis:
- Inclusion/Exclusion Based on Age: Distribution justified scientifically
- Inclusion plan is appropriate.

Vertebrate Animals:

Not Applicable (No Vertebrate Animals)

Biohazards:

Acceptable

- Blood spot data will be collected - no safety concerns.

Applications from Foreign Organizations:

Select Agents:

Not Applicable (No Select Agents)

Resource Sharing Plans:

Acceptable

Authentication of Key Biological and/or Chemical Resources:

Not Applicable (No Relevant Resources)

Budget and Period of Support:

Recommend as Requested

CRITIQUE 2

Significance: 1
Investigator(s): 2
Innovation: 1
Approach: 3
Environment: 1

Overall Impact: Young people with HIV (YPWH) are disproportionately impacted by HIV/AIDS, especially in Zambia where the proposed study will be conducted. The challenges of HIV treatment within this group are exacerbated by substance use. The goal of the study is to develop and pilot a multi-component, mHealth intervention to promote HIV self-management and reduce substance use. The impact of the study is high. The proposed project extends the team's previous research by creating an mHealth version of an already evidence-based intervention to increase access and adherence to HIV care and reduce substance use. Significance and innovation are high. The investigative team is strong with relevant expertise in mHealth interventions, HIV prevention, working with YPWH in Zambia, and the use of the MOST framework. The approach has some very strong aspects such as compelling preliminary data, engagement of a community advisory board, and use of multiple methods to adapt the intervention. Most of the weaknesses were minor. However, two weakness create some concern. First, the control group condition was not well described. Additional details are needed to increase the confidence in the retention and data collection capabilities without ongoing contacts. Second, the intervention is designed to address both HIV self-management and substance use, but participants will be asked to choose one or the other as a focus at the start of the intervention. Additional explanation of ordering and comparison the effects of timing of intervention components. In conclusion, despite the weakness in the approach, the potential overall impact of this project remains high because it may enhance delivery, sustainability, and implementation towards ending the HIV epidemic.

1. Significance:

Strengths

- The HC via mHealth may enhance delivery, sustainability, and implementation towards ending the HIV epidemic.
- The potential for scale up and mass dissemination to other contexts like Zambia is high.

Weaknesses

- None noted.

2. Investigator(s):

Strengths

- History of collaboration and productivity…PI has worked with Zambian co-Is to develop and test mHealth interventions to improve medication adherence among patients with chronic diseases.
- The PI has demonstrated expertise in adolescent and young adult health, HIV prevention, intervention development, and use of the MOST framework.
- Strong team of experts representing other key components for the successful completion of this proposal including mHealth intervention development, qualitative research, and substance use within the Zambian context.

Weaknesses

- MPI plan not well justified… unclear what MPI MacDonell, who has no history working with Zambian partners, adds to the investigative team.

3. Innovation:

Strengths

- Use of the MOST framework to identify the most effective intervention component or combination of components that addresses self-management and substance use among YPWH in Zambia.
- Concurrently addressing HIV self-care management and substance use among YPWH in Zambia is distinct from other approaches to address HIV treatment adherence among this group and in this setting.
- The proposed mHealth intervention will allow for an adaptive centralized delivery model, to address barriers to clinic-based delivery and promote future scalability and sustainment across clinics; this addresses challenges in earlier trials.
- Clear description on how findings from Phase 1 will contribute to subsequent Phases.

Weaknesses

- The use of multiple methods of intervention tailoring in not novel.

4. Approach:

Strengths

- The experimental design is comprehensive and cohesively covers all aspects of intervention adaptation.
- Preliminary data of in-person version of the intervention and partnerships with large hospitals provides evidence that the team can successfully access priority population and execute the research.
- Use of the Multiphase Optimization SMOST) framework to identify the most effective.
- Use of multiple and mixed methods to adapt intervention as well as assess feasibility, acceptability, and preliminary efficacy.

Weaknesses

- Smart phone ownership was not an inclusion criteria. Investigators state that a smart phone will be provided to those who do not have one. However, smart phone costs were not included in the budget justification nor was the process of securing phones for YPWH discussed in the

proposal. Given smart phones are a core feature of this intervention, more information is needed to determine the extent to which all YPWH can access the intervention.

- Control group condition not well described. Additional detailed needed to determine how retention and data collection will occur with them in the absence of ongoing contact.
- Intervention participants will be asked to focus on HIV self-management OR substance use reduction. Rationale for doing one and not both is not discussed.
- Compensation is not listed for participants who complete exit interviews.

5. Environment:

Strengths

- Collectively, UMass Chan School of Medicine, Florida State University and the University of Zambia have the resources and infrastructure needed to support the proposed study.
- The presence of the Division of Health Informatics and Implementation Sciences, led by Dr. Gerber (co-I), the Adolescent and Emerging Adult Health program and the UTH Pediatric Centre of Excellence guarantee that expertise will be available to provide appropriate resources for the project.

Weaknesses

- Detailed descriptions of UMass departments and individuals not included in the grant overshadows aspects of the environment that were relevant to the current study.

Study Timeline:

Strengths

- Timeline seems feasible.

Weaknesses

- None noted.

Protections for Human Subjects:

Acceptable Risks and/or Adequate Protections

- PIs go beyond what is required to also include mental health support for participants.

Data and Safety Monitoring Plan (Applicable for Clinical Trials Only):

Acceptable

- o DSMP is appropriate.

Inclusion Plans:

- Sex/Gender: Distribution justified scientifically
- Race/Ethnicity: Distribution justified scientifically
- For NIH-Defined Phase III trials, Plans for valid design and analysis:
- Inclusion/Exclusion Based on Age: Distribution justified scientifically

- Emerging/young adults (ages 18-24) are characterized by increasing independence, risk-taking behaviors, identity exploration, and changing social supports. The challenges during the transition to adulthood may extend to poor HIV management behaviors and risk-taking, including medication adherence and retention in HIV care, as well as risky sexual behavior. We choose to study 18-24-years old Zambian emerging adults because of 1) very high levels of sexual risk behaviors and substance use; and 2) high rates of HIV/STI infections in this subgroup.

Vertebrate Animals:
Not Applicable (No Vertebrate Animals)

Biohazards:
Not Applicable (No Biohazards)

Applications from Foreign Organizations:
Justified

Select Agents:
Not Applicable (No Select Agents)

Resource Sharing Plans:

Authentication of Key Biological and/or Chemical Resources:
Not Applicable (No Relevant Resources)

Budget and Period of Support:
Recommend as Requested

CRITIQUE 3

Significance: 2
Investigator(s): 1
Innovation: 2
Approach: 2
Environment: 1

Overall Impact: The proposed study seeks to develop and pilot a multi-component, mobile health (mHealth) intervention to promote HIV self-management and reduce substance use among young people with HIV in Zambia. It will adapt a 4-session, in-person intervention, Health Choices (HC), for mHealth (mHC) to increase access and delivery of HC, and will develop and pilot test Motivational Text Messaging (MTM) to enhance the impact of mHC. By combining mHealth and MTM, the study aims to develop an optimized mHealth intervention to support YPWH's self-management of HIV, which can be tested in future larger efficacy trials. The investigative team has a strong record of collaboration and relevant experience, the environment is suitable for the research, the approach is rigorous and considers challenges and alternative strategies, and the methods used are innovative.

1. Significance:

Strengths

- Given the high prevalence of both HIV and substance use in Sub-Saharan Africa, including in Zambia, and the disproportionate impact of HIV on young people, a mobile health (mHealth) culturally-tailored intervention targeting HIV and substance use reduction among a vulnerable population in a highly-affected region contributes to the relevance and significance of the proposed research. Delivering in-person interventions in resource-limited settings may be even more challenging; therefore mHealth may increase access.
- Engagement, and implementation.
- There is a lack of evidence-based interventions for substance using youth in Zambia. The HC intervention conducted by the investigators appears to be the only intervention for young people with HIV that has shown improvements in viral load and substance use.
- There is a scientific and theoretical premise for the study, and the intervention uses the Information-Motivation-Behavioral Skills (IMB) model and Socioecological Model (SEM) as conceptual frameworks.

Weaknesses

- None noted.

2. Investigator(s):

Strengths

- The investigative team has a strong existing collaboration and experience conducting high-level research studies, particularly on HIV prevention, adolescent health risk behavior, implementation of youth evidence-based interventions, including eHealth and mHealth interventions. They have conducted HIV prevention studies in Thailand, the Bahamas, China, and the US and have an extensive publication record.

Weaknesses

- None noted.

3. Innovation:

Strengths

- The proposal demonstrates innovation by adapting an existing in-person intervention, Healthy Choices (HC), to a mHealth format (mHC).
- The use of Computerized Intervention Authoring System (CIAS) as a delivery platform adds technological innovation to the study. CIAS features an interactive 3-D narrator, clear and relevant graphics, and audio to mimic the conversational nature of person-delivered counseling.
- The incorporation of tailored Motivational text messaging (MTM) that are easily programmed and automated to provide ongoing support for self-management of health behavior of users receiving the intervention to enhance the impact is novel.

Weaknesses

- Since the HC intervention has already shown success, adapting it to an mHealth format is moderately innovative.

4. Approach:

Strengths

- The proposed study is rigorous in terms of intervention adaptation, data collection methods, data management, and analysis.
- Discussion of other mHealth interventions in the strategy adds rigor, including the intervention co-led by MPI Dr. MacDonell. The approach is strengthened by prior research conducted by the Zambian team, which has extensive experience in HIV and substance use research with young people with HIV. The finding that youth were very enthusiastic about CIAS is promising. The proposal includes a discussion of potential problems and alternative strategies.
- The investigators have already tailored MI in the intervention for young people with HIV to address stigma. Consistent with MI, mHealth intervention sessions will be unique as the participant will be able to choose to receive content according to individual needs, level of motivation, and goals.
- Using CIAS that allows the researcher to directly edit the content for new settings without a software programmer is a strength.

Weaknesses

- It is not clear whether the participants need to speak all three languages (eligibility criteria state “English, Nyanja, and Bemba” rather than “English, Nyanja, or Bemba” so this is unclear.
- Minor: It is not clear how the n = 170 in the planned inclusion enrollment report is calculated.

5. Environment:

Strengths

- The three collaborating institutions provide a supportive research environment within which to conduct the proposed study.

Weaknesses

- None noted.

Study Timeline:

Strengths

- Timeline is feasible and well justified.

Weaknesses

- None noted.

Protections for Human Subjects:

Acceptable Risks and/or Adequate Protections

- Potential risks and protections adequately described.

Data and Safety Monitoring Plan (Applicable for Clinical Trials Only):

Acceptable

- Plan is acceptable.

Inclusion Plans:

- Sex/Gender: Distribution justified scientifically
- Race/Ethnicity: Distribution justified scientifically
- For NIH-Defined Phase III trials, Plans for valid design and analysis: Not applicable
- Inclusion/Exclusion Based on Age: Distribution justified scientifically
- Acceptable.

Vertebrate Animals:
Not Applicable (No Vertebrate Animals)

Biohazards:
Not Applicable (No Biohazards)

Applications from Foreign Organizations:
Justified

Select Agents:
Not Applicable (No Select Agents)

Resource Sharing Plans:
Not Applicable (No Relevant Resources)

Authentication of Key Biological and/or Chemical Resources:
Not Applicable (No Relevant Resources)

Budget and Period of Support:
Recommend as Requested

THE FOLLOWING SECTIONS WERE PREPARED BY THE SCIENTIFIC REVIEW OFFICER TO SUMMARIZE THE OUTCOME OF DISCUSSIONS OF THE REVIEW COMMITTEE, OR REVIEWERS' WRITTEN CRITIQUES, ON THE FOLLOWING ISSUES:

PROTECTION OF HUMAN SUBJECTS: ACCEPTABLE

INCLUSION OF WOMEN PLAN: ACCEPTABLE

INCLUSION OF MINORITIES PLAN: ACCEPTABLE

INCLUSION ACROSS THE LIFESPAN: ACCEPTABLE

COMMITTEE BUDGET RECOMMENDATIONS: The budget was recommended as requested.

Footnotes for 1R34DA059935-01; PI Name: Wang, Bo

NIH has modified its policy regarding the receipt of resubmissions (amended applications).See Guide Notice NOT-OD-18-197 at https://grants.nih.gov/grants/guide/notice-files/NOT-OD-18-197.html. The impact/priority score is calculated after discussion of an application by averaging the overall scores (1-9) given by all voting reviewers on the committee and multiplying by 10. The criterion scores are submitted prior to the meeting by the individual reviewers assigned to an application, and are not discussed specifically at the review meeting or calculated into the overall impact score. Some applications also receive a percentile ranking. For details on the review process, see http://grants.nih.gov/grants/peer_review_process.htm#scoring.

MEETING ROSTER

Interventions to Prevent and Treat Addictions Study Section
Risk, Prevention and Health Behavior Integrated Review Group
CENTER FOR SCIENTIFIC REVIEW
IPTA
06/22/2023 - 06/23/2023

Notice of NIH Policy to All Applicants: Meeting rosters are provided for information purposes only. Applicant investigators and institutional officials must not communicate directly with study section members about an application before or after the review. Failure to observe this policy will create a serious breach of integrity in the peer review process, and may lead to actions outlined in NOT-OD-22-044 at https://grants.nih.gov/grants/guide/notice-files/NOT-OD-22-044.html, including removal of the application from immediate review.

CHAIRPERSON(S)

PIPER, MEGAN E, PHD
PROFESSOR
DEPARTMENT OF MEDICINE
UNIVERSITY OF WISCONSIN-MADISON
MADISON, WI 53711

MEMBERS

ALETRARIS, LYDIA, PHD *
ASSOCIATE RESEARCH SCIENTIST
SCHOOL OF SOCIAL WORK
UNIVERSITY OF GEORGIA
ATHENS, GA 30602

BERNSTEIN, STEVEN L, MD
PROFESSOR
DEPARTMENT OF EMERGENCY MEDICINE
GEISEL SCHOOL OF MEDICINE AT DARTMOUTH
LEBANON, NH 03756

BINSWANGER, INGRID A, MD, MPH
SENIOR CLINICIAN INVESTIGATOR
INSTITUTE FOR HEALTH RESEARCH
KAISER PERMANENTE
AURORA, CO 80014

BRICKER, JONATHAN B, PHD
PROFESSOR
DIVISION OF PUBLIC HEALTH SCIENCES
FRED HUTCHINSON CANCER RESEARCH CENTER
UNIVERSITY OF WASHINGTON
SEATTLE, WA 98109

CLAUS, ERIC D, PHD
ASSOCIATE PROFESSOR
DEPARTMENT OF BIOBEHAVIORAL HEALTH
THE PENNSYLVANIA STATE UNIVERSITY
UNIVERSITY PARK, PA 16802

FENDRICH, MICHAEL, PHD *
SCIENTIFIC DIRECTOR
ADVOCATE AURORA RESEARCH INSTITUTE
MILWAUKEE, WI 53211

GAINES, TOMMI LYNN, DRPH *
ASSOCIATE PROFESSOR
DIVISION OF GLOBAL PUBLIC HEALTH
DEPARTMENT OF MEDICINE
SCHOOL OF MEDICINE
UNIVERSITY OF CALIFORNIA, SAN DIEGO
LA JOLLA, CA 92093

GARRISON, KATHLEEN A, PHD
ASSISTANT PROFESSOR
DEPARTMENT OF PSYCHIATRY
YALE SCHOOL OF MEDICINE
NEW HAVEN, CT 06510

GRYCZYNSKI, JAN, PHD
SENIOR RESEARCH SCIENTIST
FRIENDS RESEARCH INSTITUTE
BALTIMORE, MD 21201

HAGLE, HOLLY *
ASSOCIATE RESEARCH PROFESSOR
MISSOURI CENTER FOR ADDICTION RESEARCH AND ENGAGEMENT
COLLEGE OF ARTS AND SCIENCE
UNIVERSITY OF MISSOURI
KANSAS CITY, MO 64110

JAGER, JUSTIN O, PHD *
ASSOCIATE PROFESSOR
COLLEGE OF HEALTH SOLUTIONS
THE SANFORD SCHOOL
ARIZONA STATE UNIVERSITY
TEMPE, AZ 85281

KELLEY, MICHELLE L, PHD *
PROFESSOR AND CHAIR
DEPARTMENT OF PSYCHOLOGY
COLLEGE OF SCIENCES
OLD DOMINION UNIVERSITY
NORFOLK, VA 23322

KOWALCHUK, ALICIA ANN, DO *
ASSOCIATE PROFESSOR
BAYLOR COLLEGE OF MEDICINE
HOUSTON, TX 77030

LANGDON, KIRSTEN JOHNSON, PHD *
ASSISTANT PROFESSOR
DEPARTMENT OF BEHAVIORAL AND SOCIAL SCIENCES
DEPARTMENT OF PSYCHIATRY AND HUMAN BEHAVIOR
BROWN UNIVERSITY
PROVIDENCE, RI 02912

LEDGERWOOD, DAVID M, PHD
PROFESSOR
DEPARTMENT OF PSYCHIATRY
AND BEHAVIORAL NEUROSCIENCES
SCHOOL OF MEDICINE
WAYNE STATE UNIVERSITY
DETROIT, MI 48201

MATHEW, AMANDA R, PHD *
ASSISTANT PROFESSOR
DEPARTMENT OF PREVENTIVE MEDICINE
RUSH UNIVERSITY
CHICAGO, IL 60612

MCHUGH, REBECCA KATHRYN, PHD
ASSOCIATE PROFESSOR
DEPARTMENT OF PSYCHIATRY
HARVARD MEDICAL SCHOOL
BELMONT, MA 02478

MCNEELY, JENNIFER, MD
ASSOCIATE PROFESSOR
DEPARTMENTS OF POPULATION HEALTH AND MEDICINE
SCHOOL OF MEDICINE
NEW YORK UNIVERSITY
NEW YORK, NY 10016

MCPHERSON, STERLING M, PHD
PROFESSOR AND DIRECTOR
PROGRAM OF EXCELLENCE IN ADDICTIONS RESEARCH
DEPARTMENT OF COMMUNITY AND BEHAVIORAL HEALTH
ELSON S. FLOYD COLLEGE OF MEDICINE
WASHINGTON STATE UNIVERSITY
SPOKANE, WA 99210

MILLS, SARAH, PHD, MPH *
ASSISTANT PROFESSOR
DEPARTMENT OF HEALTH BEHAVIOR
GILLINGS SCHOOL OF GLOBAL PUBLIC HEALTH
UNIVERSITY OF NORTH CAROLINA
CHAPEL HILL, NC 27599

MONTGOMERY, LATRICE, PHD
ASSOCIATE PROFESSOR
DEPARTMENT OF PSYCHIATRY AND
BEHAVIORAL NEUROSCIENCE
COLLEGE OF MEDICINE
UNIVERSITY OF CINCINNATI
CINCINNATI, OH 45229

MUMBA, MERCY N, PHD
ASSOCIATE PROFESSOR
CAPSTONE COLLEGE OF NURSING
THE UNIVERSITY OF ALABAMA
TUSCALOOSA, AL 35401

NAPPER, LUCY ELIZABETH, PHD *
ASSOCIATE PROFESSOR
DEPARTMENT OF PSYCHOLOGY
COLLEGE OF ARTS AND SCIENCES
LEHIGH UNIVERSITY
BETHLEHEM, PA 18015

POWELL, TERRINIEKA WILLIAMS, PHD *
ASSOCIATE PROFESSOR
POPULATION, FAMILY AND REPRODUCTIVE HEALTH
BLOOMBERG SCHOOL OF PUBLIC HEALTH
JOHNS HOPKINS UNIVERSITY
BALTIMORE, MD 21205

RAIFF, BETHANY R, PHD *
PROFESSOR
DEPARTMENT OF PSYCHOLOGY
COLLEGE OF SCIENCE AND MATHEMATICS
ROWAN UNIVERSITY
GLASSBORO, NJ 08028

SADASIVAM, RAJANI, PHD
PROFESSOR
DIVISION OF HEALTH AND IMPLEMENTATION SCIENCE
DEPARTMENT OF POPULATION AND QUANTITATIVE
HEALTH SCIENCES
UNIVERSITY OF MASSACHUSETTS MEDICAL SCHOOL
WORCESTER, MA 01605

SARAIYA, TANYA CHANDRESH, PHD *
ASSISTANT PROFESSOR
DEPARTMENT OF ALCOHOL & SUBSTANCE USE STUDIES
GRADUATE SCHOOL OF APPLIED AND PROFESSIONAL
PSYCHOLOGY
RUTGERS UNIVERSITY
PISCATAWAY, NJ 08854

SHEFFER, CHRISTINE ELIZABETH, PHD
PROFESSOR
DEPARTMENT OF HEALTH BEHAVIOR
ROSWELL PARK COMPREHENSIVE CANCER CENTER
BUFFALO, NY 14263

STATON, MICHELE, PHD
PROFESSOR
DEPARTMENT OF BEHAVIORAL SCIENCES
COLLEGE OF MEDICINE
UNIVERSITY OF KENTUCKY
LEXINGTON, KY 40536

STORMSHAK, ELIZABETH A, PHD
PROFESSOR
COLLEGE OF EDUCATION
PREVENTION SCIENCE INSTITUTE
UNIVERSITY OF OREGON
EUGENE, OR 97403

VILARDAGA, ROGER, PHD
ASSOCIATE PROFESSOR
DEPARTMENT OF PSYCHIATRY AND BEHAVIORAL SCIENCES
SCHOOL OF MEDICINE
DUKE UNIVERSITY
DURHAM, NC 27710

WATKINS, KATHERINE E, MD, MSHS *
SENIOR SCIENTIST
HEALTH CARE DIVISION
RAND CORPORATION
SANTA MONICA, CA 90407

WELSH, JUSTINE WITTENAUER, MD *
DIRECTOR OF THE EMORY HEALTHCARE ADDICTION SERVICES
DEPARTMENT OF PSYCHIATRY AND BEHAVIORAL SCIENCES
SCHOOL OF MEDICINE
EMORY UNIVERSITY
ATLANTA, GA 30307

YI, RICHARD, PHD
PROFESSOR AND DIRECTOR
DEPARTMENT OF PSYCHOLOGY
UNIVERSITY OF KANSAS
LAWRENCE, KS 66045

MAIL REVIEWER(S)

D'SOUZA, DEEPAK CYRIL, MD
PROFESSOR
DEPARTMENT OF PSYCHIATRY
YALE UNIVERSITY
WEST HAVEN, CT 06516

SCIENTIFIC REVIEW OFFICER

VIDAL, SARAH, PHD
SCIENTIFIC REVIEW OFFICER
CENTER FOR SCIENTIFIC REVIEW
NATIONAL INSTITUTES OF HEALTH
BETHESDA, MD 20892

EXTRAMURAL SUPPORT ASSISTANT

AMARE, MERON ERMIAS
LEAD EXTRAMURAL SUPPORT ASSISTANT
CENTER FOR SCIENTIFIC REVIEW
NATIONAL INSTITUTES OF HEALTH
BETHESDA, MD 20892

* Temporary Member. For grant applications, temporary members may participate in the entire meeting or may review only selected applications as needed.

Consultants are required to absent themselves from the room during the review of any application if their presence would constitute or appear to constitute a conflict of interest.
